# Identification of Transcriptional Signatures of Colon Tumor Stroma by a Meta-Analysis

**DOI:** 10.1155/2019/8752862

**Published:** 2019-05-02

**Authors:** Md. Nazim Uddin, Mengyuan Li, Xiaosheng Wang

**Affiliations:** ^1^Biomedical Informatics Research Lab, School of Basic Medicine and Clinical Pharmacy, China Pharmaceutical University, Nanjing 211198, China; ^2^Cancer Genomics Research Center, School of Basic Medicine and Clinical Pharmacy, China Pharmaceutical University, Nanjing 211198, China; ^3^Big Data Research Institute, China Pharmaceutical University, Nanjing 211198, China

## Abstract

**Background:**

The tumor stroma plays pivotal roles in influencing tumor growth, invasion, and metastasis. Transcriptional signatures of colon tumor stroma (CTS) are significantly associated with prognosis of colon cancer. Thus, identification of the CTS transcriptional features could be useful for colon cancer diagnosis and therapy.

**Methods:**

By a meta-analysis of three CTS gene expression profiles datasets, we identified differentially expressed genes (DEGs) between CTS and colon normal stroma. Furthermore, we identified the pathways, upstream regulators, and protein-protein interaction (PPI) network that were significantly associated with the DEGs. Moreover, we analyzed the enrichment levels of immune signatures in CTS. Finally, we identified CTS-associated gene signatures whose expression was significantly associated with prognosis in colon cancer.

**Results:**

We identified numerous significantly upregulated genes (such as* CTHRC1*,* NFE2L3*,* SULF1*,* SOX9*,* ENC1*, and* CCND1*) and significantly downregulated genes (such as* MYOT*,* ASPA*,* KIAA2022*,* ARHGEF37*,* BCL-2*, and* PPARGC1A*) in CTS versus colon normal stroma. Furthermore, we identified significantly upregulated pathways in CTS that were mainly involved in cellular development, immune regulation, and metabolism, as well as significantly downregulated pathways in CTS that were mostly metabolism-related. Moreover, we identified upstream TFs (such as SUZ12, NFE2L2, RUNX1, STAT3, and SOX2), kinases (such as MAPK14, CSNK2A1, CDK1, CDK2, and CDK4), and master metabolic transcriptional regulators (MMTRs) (such as HNF1A, NFKB1, ZBTB7A, GATA2, and GATA5) regulating the DEGs. We found that CD8+ T cells were more enriched in CTS than in colon normal stroma. Interestingly, we found that many of the DEGs and their regulators were prognostic markers for colon cancer, including* CEBPB*,* PPARGC1*,* STAT3*,* MTOR*,* BCL2*,* JAK2*, and* CDK1*.

**Conclusions:**

The identification of CTS-specific transcriptional signatures may provide insights into the tumor microenvironment that mediates the development of colon cancer and has potential clinical implications for colon cancer diagnosis and treatment.

## 1. Background

The tumor stroma is an important component of the tumor microenvironment (TME) and plays key roles in the tumor development [1]. Stromal cells are composed of many different types of cells, including vascular endothelial cells, pericytes, adipocytes, fibroblasts, osteoblasts, chondrocytes, extracellular matrix (ECM), and bone-marrow mesenchymal stromal cells [2]. The tumor stroma can promote ECM remodeling, cellular migration, neoangiogenesis, invasion, immunosurveillance evasion, and drug resistance of tumors [3]. Colorectal cancer (CRC) is the fourth most common cancer and a leading cause of cancer mortality worldwide [4]. Transcriptional signatures of CRC stromal cells have been associated with poor prognosis in CRC [5]. Isella et al. demonstrated that the gene signatures of CRC stromal cells (cancer-associated fibroblasts, leukocytes, and endothelial cells) were significantly upregulated in the stem/serrated/mesenchymal transcriptional subtype of CRC which had a poor prognosis [6]. Calon et al. showed that the CRC stromal transcriptional signatures correlated with disease relapse [5]. These prior studies exhibited the significant roles of tumor stroma in CRC growth, invasion, and metastasis.

In this study, we performed a meta-analysis of three colon tumor stromal transcriptome datasets using the bioinformatics approach. We identified differentially expressed genes (DEGs) between colon tumor stroma (CTS) and normal stroma. On the basis of these DEGs, we identified their associated pathways, upstream regulators, and protein-protein interaction (PPI) network and certain prognostic markers that were associated with survival of colon cancer patients. We also analyzed the enrichment levels of immune signatures in CTS. This study provides insights into CTS molecular features that could have clinical implications for colon cancer diagnosis and treatment.

## 2. Methods

### 2.1. Datasets

We searched the NCBI Gene Expression Omnibus (GEO) database (https://www.ncbi.nlm.nih.gov/geo/) using the keywords “colon cancer,” “stroma,” and “tumor stroma” and identified three CTS gene expression profiles datasets (GSE31279, GSE35602, and GSE46824) [7–9]. In survival analyses, we used the TCGA colon cancer dataset (https://portal.gdc.cancer.gov/) and a SurvExpress (http://bioinformatica.mty.itesm.mx/SurvExpress) built-in dataset (colon metabase) [10]. A summary of these datasets is shown in Supplementary Table S1.

### 2.2. Identification of DEGs between CTS and Normal Stroma

We used the web tool Network Analyst [11] to identify the DEGs between CTS and normal stroma. The ComBat method [12] in the tool was utilized to remove batch effects from the three CTS datasets (Supplementary Figure S1). Each individual dataset was normalized by base-2 log transformation and quantile normalization, and the R package “limma” was utilized to identify the DEGs between CTS and normal stroma. A meta-analysis of the three datasets was performed using Cochran's combination test [13]. The false discovery rate (FDR), calculated by the Benjamini–Hochberg method [14], was used to adjust for multiple tests. We determined the DEGs with a threshold of absolute combined effect size (ES) >0.82 and FDR<0.05.

### 2.3. Gene-Set Enrichment Analysis

We performed gene-set enrichment analysis of the DEGs by GSEA [15]. The KEGG pathways significantly associated with the upregulated and the downregulated DEGs were identified (FDR < 0.05), respectively.

### 2.4. Identification of Transcription Factors (TFs), Kinases, and Master Metabolic Transcriptional Regulators (MMTRs) That Are Significantly Associated with the DEGs

To link gene expression signatures to upstream cell signaling networks, we used eXpression2Kinases [16] to identify the upstream TFs and kinases that regulate the DEGs and utilized iRegulon [17] to identify the MMTRs of the DEGs.

### 2.5. Identification of PPI Network of the DEGs

We employed Network Analyst [11] to construct a PPI network of the DEGs [11]. Two types of modules (function-first modules and connection-first modules) of the PPI network were extracted. The function-first modules (FFMs) were constructed by pathway enrichment analysis and the connection-first modules (CFMs) were identified by the random walk-based algorithm [18].

### 2.6. Comparison of the Enrichment Levels of CD8+ T Cells between Two Classes of Samples

The enrichment level of CD8+ T cells in a sample was evaluated by the expression level of* CD8A*. We compared the enrichment levels of CD8+ T cells between two groups of samples using Student's* t*-test.

### 2.7. Identification of DEGs between High-Stroma-Content and Low-Stroma-Content TCGA Colon Cancer Samples

We used ESTIMATE [19] to quantify the intratumoral stromal content (stroma score) of TCGA colon cancer samples. We identified the DEGs between high-stroma-content (stroma score > median) and low-stroma-content (stroma score < median) tumors using Student's* t*-test.

### 2.8. Survival Analyses

We compared the overall survival (OS) and the disease-free survival (DFS) of colon cancer patients classified based on gene expression levels (expression levels > median versus expression levels < median). Kaplan-Meier survival curves were used to show the survival differences, and the log-rank test was utilized to evaluate the significance of survival differences. The individual prognostic genes were identified and were fitted in a multivariate Cox regression model. SurvExpress [10] was used for the multivariate survival analysis.

## 3. Results

### 3.1. Identification of DEGs between CTS and Normal Stroma

**Figure 1:**
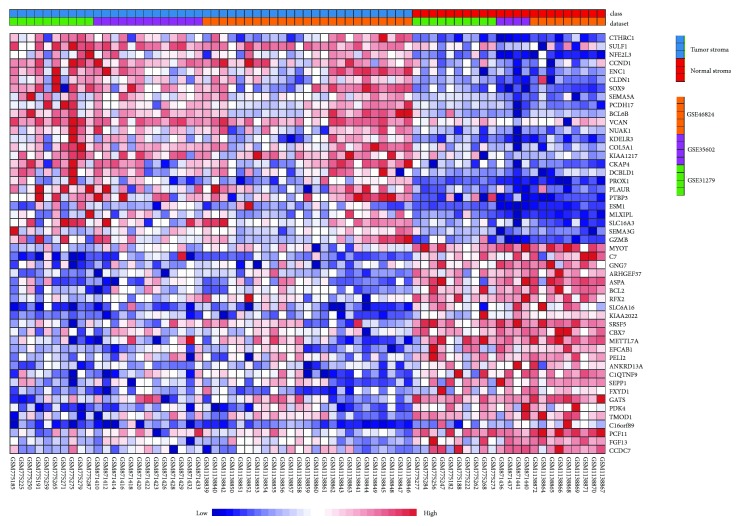
Gene expression pattern of the top 25 upregulated and top 25 downregulated genes in colon tumor stroma (CTS) relative to colon normal stroma ranked on the basis of the combined effect size (ES) identified by Network Analyst [11].

We identified 694 DEGs between CTS and normal stroma by the meta-analysis. These DEGs included 295 downregulated and 399 upregulated genes in CTS (Supplementary Tables S2 and S3).  Figure 1 shows the top 25 upregulated and top 25 downregulated genes in CTS ranked on the basis of the combined ES (the detailed results of statistical analysis for the top 10 upregulated and top 10 downregulated genes in CTS are shown in Supplementary Tables S4).* CTHRC1,* a gene involved in vascular remodeling, bone formation, and developmental morphogenesis, was upregulated in CTS with the highest ES. It has been shown that* CTHRC1* could promote human CRC cell proliferation and invasion by activating Wnt/PCP signaling [20]. This gene also plays an important role in promoting ovarian cancer cell adhesion, migration, and metastasis through the activation of integrin *β*3/FAK signaling [21].* NFE2L3*, a gene regulating the cell cycle progression in colon cancer [22], was upregulated in CTS with the second highest ES. Interestingly, both* CTHRC1* and* NFE2L3* have been indicated as useful biomarker candidates for CRC diagnosis because of their overexpression in adenomas and CRC relative to normal tissue [23].* SULF1*, whose expression in tumor stroma is a prognostic marker in advanced pancreatic cancer [24], was upregulated in CTS with the third highest ES. The overexpression of this gene has been associated with a poor prognosis in urothelial carcinoma [25].* SOX9*, the gene upregulated in CTS with the fourth highest ES, has been shown to be overexpressed in CRC and its overexpression was an independent adverse prognosticator in CRC [26]. Some other genes upregulated in CTS have been demonstrated to be overexpressed in CRC and their expression was negatively associated with CRC prognosis, such as* ENC1*,* CCND1*, V*CAN*,* SEMA5A*, and* NOS3* [27–31]. Interestingly, both* PCDH17* and* BCL6B* were upregulated in CTS, while they had reduced expression in CRC [32, 33]. It indicates that* PCDH17* and* BCL6B* could be specifically expressed in CTS cells but not in colon cancer cells.

Many of the significantly downregulated genes in CTS have been associated with CRC [34–37]. For example,* MYOT*,* ASPA*, and* KIAA2022* were downregulated in CRC [34], the downregulation of* ARHGEF37* was associated with a poor prognosis in CRC [35], higher expression levels of* BCL-2* were correlated with a better survival prognosis in CRC [36], and* PPARGC1A* was a negative predictor for CRC prognosis [37].

Altogether, a number of the abnormally expressed genes in CTS compared to colon normal stroma identified by the meta-analysis have been associated with CRC pathology and prognosis.

### 3.2. Identification of Pathways Significantly Associated with the DEGs

**Figure 2:**
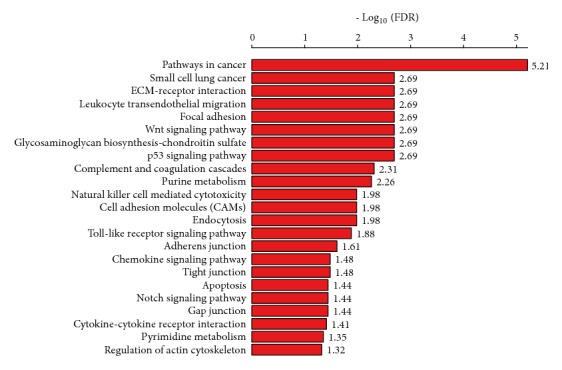
*Significantly upregulated KEGG pathways in CTS relative to colon normal stroma identified by GSEA [15]*. FDR: false discovery rate.

GSEA [15] identified 44 KEGG pathways that were significantly associated with the upregulated genes in CTS. These pathways were mainly involved in cellular development (p53 signaling, Wnt signaling, apoptosis, Notch signaling, focal adhesion, endocytosis, ECM-receptor interaction, cell adhesion molecules, adherens junction, tight junction, gap junction, and regulation of actin cytoskeleton), immune regulation (leukocyte transendothelial migration, complement and coagulation cascades, natural killer cell mediated cytotoxicity, Toll-like receptor, chemokine signaling, and cytokine-cytokine receptor interaction), and metabolism (purine metabolism and pyrimidine metabolism) (Figure 2, Supplementary Table S5). Previous studies have shown that some of these pathways were significantly associated with colon cancer [38–41]. For example, the Wnt and Notch pathways were associated with colon cancer development [38, 39]. The cytokine-cytokine receptor interaction pathway was significantly enriched in CRC [34]. The ECM and ECM-associated proteins [39], the glycosaminoglycan metabolism, and chondroitin sulfate/dermatan sulfate metabolism pathways played key roles in mediating tumor microenvironment [40, 41].

In addition, GSEA identified six KEGG pathways that were significantly associated with the downregulated genes in CTS (Supplementary Figure S2). Most of these pathways were metabolism-related, including purine metabolism, histidine metabolism, glycine, serine, and threonine metabolism, and drug metabolism-cytochrome p450. These pathways have been associated with colon and other cancers [42–44]. For example, impaired purine metabolism was associated with the progression of cancer [42]. Histidine metabolism could boost cancer therapy [43]. Cytochrome P450 enzymes were associated with the metabolism of anticancer drugs and their expression was associated with a poor prognosis in CRC patents [44].

### 3.3. Identification of Upstream TFs, Kinases, and MMTRs Significantly Associated with the DEGs

**Figure 3:**
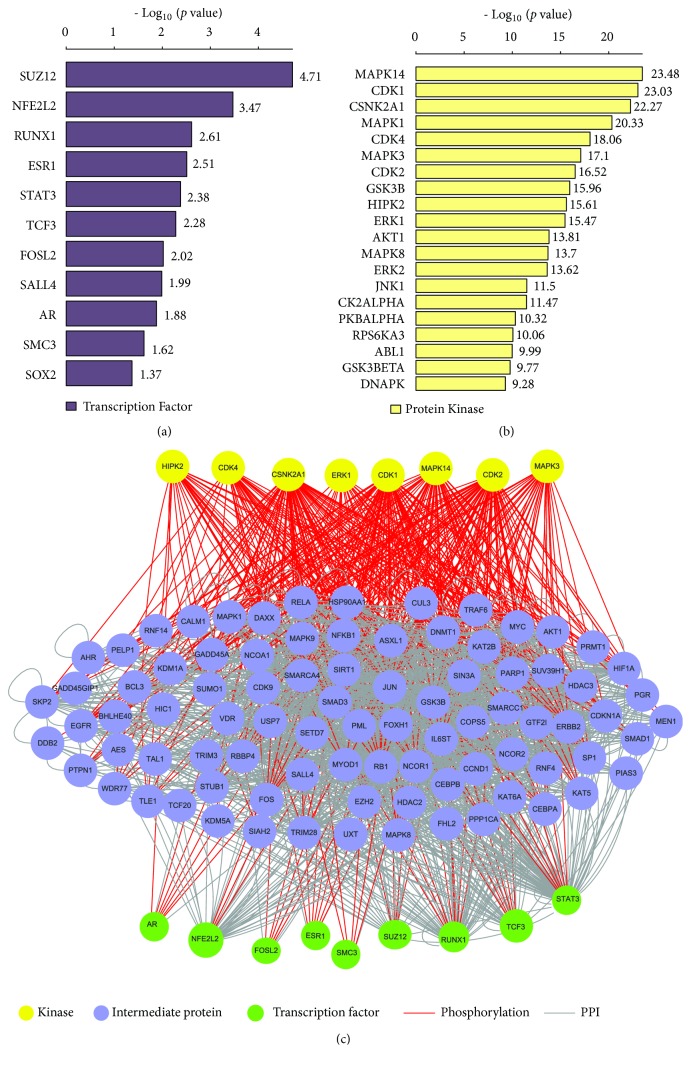
*The significant upstream transcriptional factors (TFs) and kinases that regulate the differentially expressed genes (DEGs) between CTS and colon normal stroma identified by eXpression2Kinases [16].* (a) Significant upstream TFs regulating the DEGs. (b) Significant upstream kinases regulating the DEGs. (c) A TF-kinase interaction network of the significant upstream TFs and kinases regulating the DEGs.

We identified 11 significant upstream TFs regulating the DEGs, including SUZ12, NFE2L2, RUNX1, ESR1, STAT3, TCF3, FOSL2, SALL4, AR, SMC3, and SOX2, of which the genes encoding RUNX1 and SALL4 were upregulated in CTS (Figure 3(a)). Most of these TFs have been associated with colon cancer [45–49]. For example, SUZ12 was the most significant upstream TF which could contribute to the CRC development [45]. RUNX1 mutations were associated with the CRC risk [46]. TCF3 and FOSL2 were associated with the tumorigenesis of CRC [47, 48]. The overexpression of SOX2 was associated with the progression and a poor prognosis in colon cancer [49].

Moreover, we identified 124 significant protein kinases that regulate the DEGs (Figure 3(b), Supplementary Table S6). These kinases mainly included cell cycle regulation kinases (CDKs), signaling MAP kinases (MAPKs, MAP2Ks, and MAP3Ks), and ribosomal kinases (RPS6KA1, RPS6KA3, and RPS6KA5). MAPK14 was the most significant upstream kinase negatively regulating the formation of colitis-associated colon tumors [50]. Furthermore, we constructed a TF-kinase interaction network of these TFs and kinases (Figure 3(c)). In the network, the most connected TFs included SUZ12, NFE2L2, RUNX1, STAT3, FOSL2, AR, SMC3, ESR1, and TCF3, and the most connected kinases included MAPK14, CDK1, CSNK2A1, CDK2, MAPK3, HIPK2, ERK1, and CDK4. It indicates that the cell cycle regulation may play a pivotal role in CTS.

**Figure 4:**
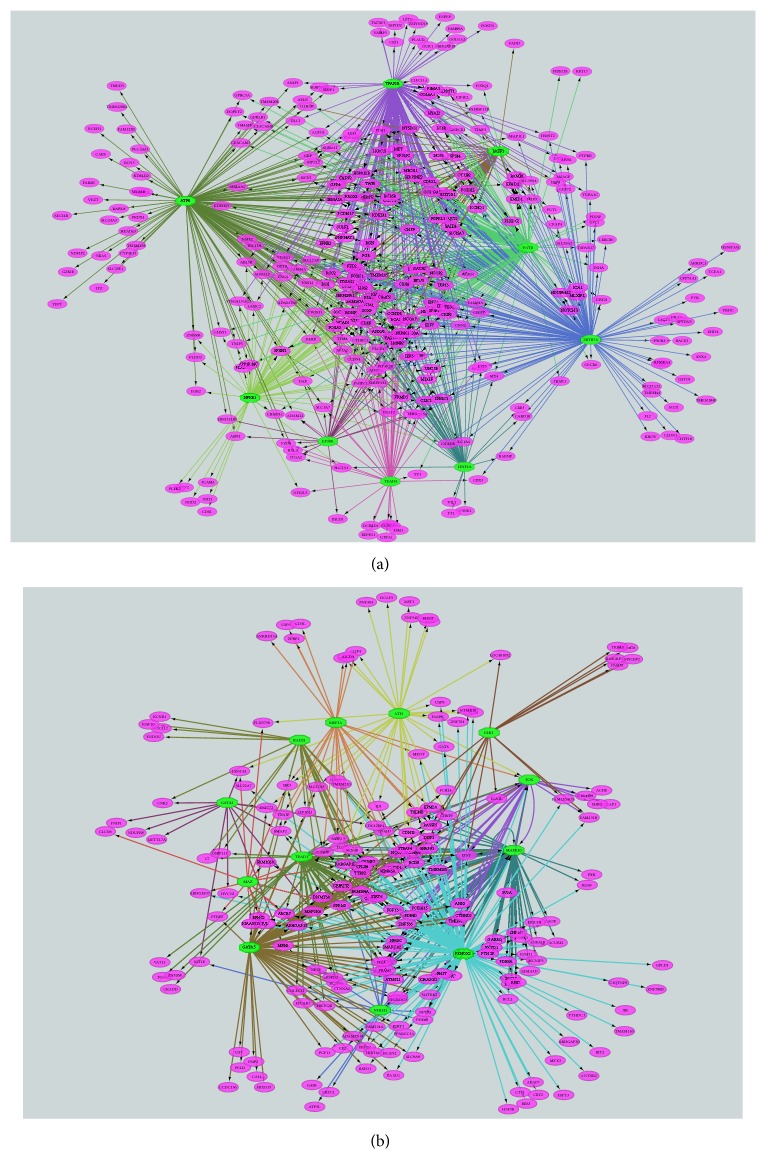
*Regulatory networks of the master metabolic transcriptional regulators (MMTRs) and their targeted differentially expressed genes (DEGs) between CTS and normal stroma identified by iRegulon [17]*. (a) Regulatory network of the MMTRs and their targeted upregulated genes in CTS. (b) Regulatory network of the MMTRs and their targeted downregulated genes in CTS. The green color octagon indicates MMTRs and purple color oval indicates DEGs.

MMTRs are interesting biomarkers and targets for metabolism-targeted cancer therapy [51]. We identified 9 (HNF1A, NFKB1, ZBTB7A, ATF6, TEAD4, TFAP2B, JAZF1, FNTB, and EP300) and 12 (PKNOX2, GATA2, MAPK10, TEAD1, TOX, MEF2A, GATA5, ELK1, MAZ, NHLH1, ATF1, and RAD21) MMTRs for the upregulated and the downregulated genes in CTS, respectively (Supplementary Table S7), and built the regulatory networks associated with these MMTRs (Figure 4). In the networks, ATF6 (activating transcription factor 6), a TF regulating unfolded protein response during endoplasmic reticulum (ER) stress, targeted 163 upregulated genes, and PKNOX2 (PBX/knotted 1 homeobox 2), which plays key roles in regulating cell proliferation, differentiation, and death, targeted 131 downregulated genes. Interestingly, two members of the GATA family of TFs (GATA2 and GATA5) were the MMTRs that regulated the downregulated genes in CTS (Figure 4(b)).

Altogether, the identification of upstream TFs, kinases, and MMTRs significantly associated with the DEGs may provide insights into the TME that mediates the development of colon cancer.

### 3.4. CD8+ T Cells Are More Enriched in CTS than in Normal Stroma

**Figure 5:**
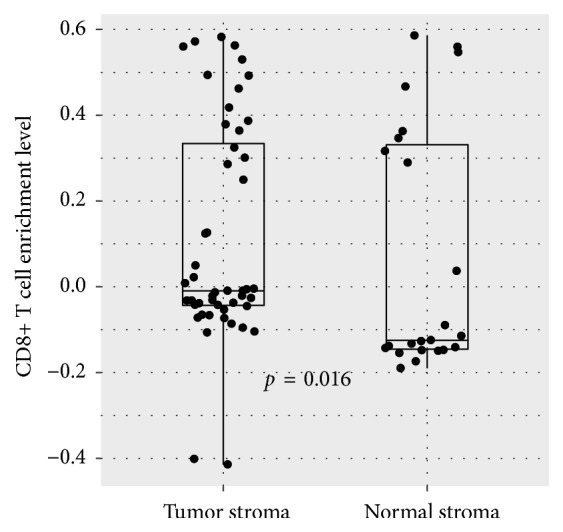
*CD8+ T cells have significantly higher enrichment levels in CTS than in colon normal stroma*. Student's* t*-test* p* value is shown.

We compared the enrichment levels of CD8+ T cells between CTS and normal stroma and found that CD8+ T cells showed significantly higher enrichment levels in CTS than in normal stroma (Student's* t*-test,* p*=0.016) (Figure 5). This suggests an antitumor immune response activity in the TME of colon cancer.

### 3.5. Identification of Prognostic Factors in Colon Cancer Based on the DEGs and Their Upstream Regulators

**Figure 6:**
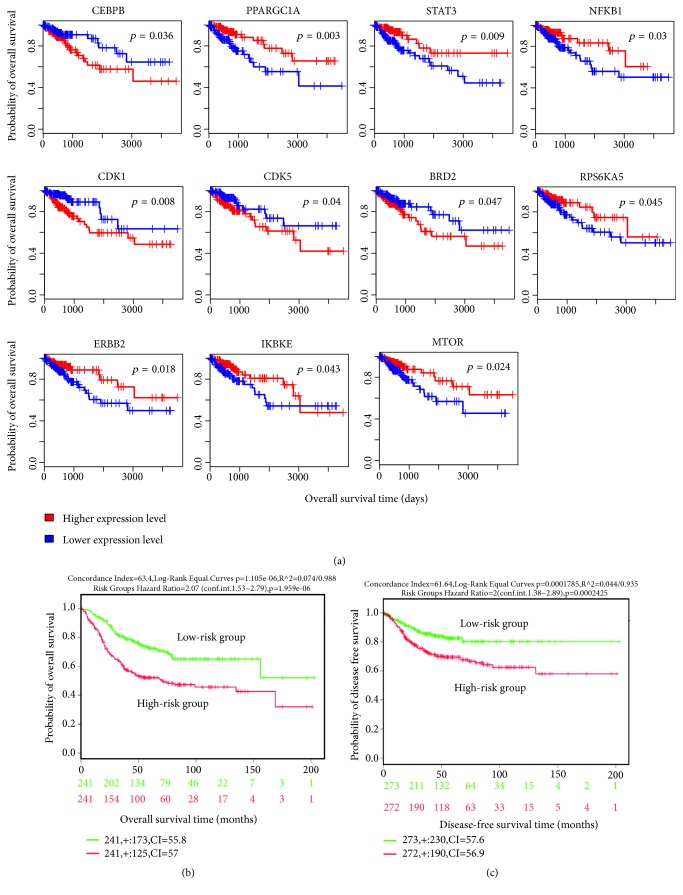
*The CTS gene signatures whose expression is associated with prognosis in colon cancer*. (a) Kaplan-Meier survival curves show the gene signatures whose expression is significantly associated with overall survival (OS) in colon cancer in the TCGA colon cancer dataset (log-rank test,* p*<0.05). (b) Multivariate Cox regression analysis shows that the OS-associated CTS gene signatures are prognostic for OS in colon cancer in a SurvExpress built-in dataset (colon metabase) [10]. (c) Multivariate Cox regression analysis shows that the DFS-associated CTS gene signatures are prognostic for DFS in colon cancer in a SurvExpress built-in dataset (colon metabase) [10]. DFS: disease-free survival.

We investigated the association between the transcriptional signatures of CTS and survival prognosis (overall survival (OS) and disease-free survival (DFS)) in the TCGA colon cancer dataset. The transcriptional signatures included the top 10 upregulated and top 10 downregulated genes in CTS on the basis of ES, 45 hub genes (≥3 degrees) from the zero-order PPI network of the DEGs (Supplementary Table S8), and the genes encoding 11 TFs, 124 kinases, and 21 MMTRs regulating the DEGs. We found that the expression of many of these transcriptional signatures was significantly associated with the survival of colon cancer patients. For example, the expression of* CEBPB*, a gene significantly upregulated in CTS and a hub node in the PPI network, had a significant negative correlation with OS in colon cancer (Figure 6(a)). The negative correlation between* CEBPB *expression and survival has also been demonstrated in other cancer types, such as high-grade serous ovarian cancer [52].* PPARGC1* was significantly downregulated in CTS and was a hub node in the PPI network, while its expression had a significant positive correlation with OS in colon cancer (Figure 6(a)).* PPARGC1A* was indicated as a tumor suppressor in colon cancer [53] and ovarian cancer [54], as well as a negative prognostic biomarker for CRC [37]. Our data indicate that the deregulation of these genes in CTS is prognostic for colon cancer patients.

Among the upstream regulators (TFs, kinases, and MMTRs) of the DEGs, the expression of* STAT3*,* RPS6KA5*,* IKBKE*,* ERBB2*,* MTOR*, and* NFKB1* had a positive correlation with OS in colon cancer, while the expression of* CDK1*,* CDK5*, and* BRD2* had a negative correlation with OS in colon cancer (Figure 6(a)). The deregulation of these genes has been associated with tumor progression in a wide variety of cancer types [55–60].

In addition, we identified 18 transcriptional signatures of CTS whose expression was significantly associated with DFS in colon cancer individually (Supplementary Figure S3). These genes included* CEBPB*,* BCL2*,* PAN2*,* NOS3*,* FTL*,* ARHGEF37*,* SMC3*,* EP300*,* JAK2*,* RPS6KA3*,* RPS6KA1*,* PRKACA*,* HIPK1*,* HIPK2*,* MAPK8*,* GSK3A*,* CLK2*, and* CDK3.* It indicates that these CTS transcriptional signatures could be biomarkers for colon cancer relapse.

Furthermore, we used the multivariate analysis to validate the association between the prognostic CTS transcriptional signatures and survival using the colon metabase data [10]. For OS analysis, a total of 482 patients were split into two groups: high-risk group (N=241) versus low-risk group (N=241) based on the prognostic index (Supplementary Figure S4A). As expected, the high-risk group had worse OS than the low-risk group (Figure 6(b)). Similarly, for DFS analysis, we divided patients into the high-risk group (N=272) and the low-risk group (N=273) based on the prognostic index (Supplementary Figure S4B) and found that the high-risk group had worse DFS compared to the low-risk group (Figure 6(c)). These results proved the prognostic value of these CTS transcriptional signatures in colon cancer.

## 4. Discussion

The tumor stroma constitutes an important component of the TME that mediates tumor growth, immune evasion, and metastasis [1]. Thus, it is important to identify molecular features in the tumor stroma. To this end, we performed a meta-analysis of three CTS transcriptome datasets for identifying CTS-associated transcriptional signatures. We identified a number of upregulated and downregulated genes in CTS compared to colon normal stroma. Furthermore, we identified upregulated and downregulated pathways significantly associated with these deregulated genes in CTS. The upregulated pathways were mainly involved in cellular development, immune regulation, and metabolism, and the downregulated pathways were mostly metabolism-related. These results revealed the abnormal alterations of cellular development, immune regulation, and metabolism pathways in CTS. We found that CD8+ T cells were more enriched in CTS than in colon normal stroma, suggesting an immune infiltration microenvironment in CTS. Furthermore, we identified numerous CTS transcriptional signatures whose expression was significantly associated with prognosis in colon cancer, such as* CEBPB*,* PPARGC1*,* STAT3*,* MTOR*,* BCL2*,* JAK2*, and* CDK1*. These transcriptional signatures are mainly involved in immune regulation (*CEBPB*,* STAT3*, and* JAK2*), metabolism (*PPARGC1* and* MTOR*), cell cycle (*CDK1*), and apoptosis (*BCL2*), suggesting that the deregulation of these pathways in CTS may contribute to the altered prognosis in colon cancer.

**Figure 7:**
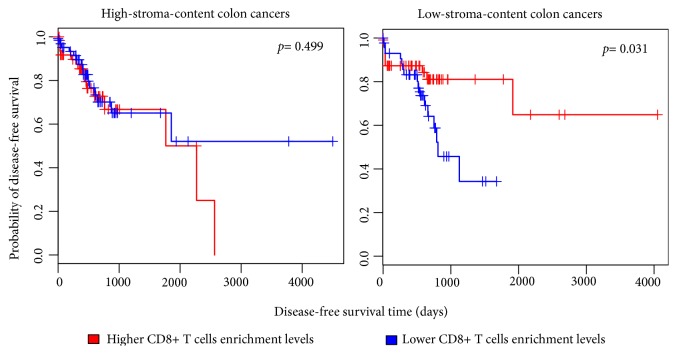
*The higher enrichment levels of CD8+ T cells were associated with better disease-free survival in the low-stroma-content colon cancers, but not in the high-stroma-content colon cancers*. ESTIMATE [19] was used to quantify the intratumoral stromal content (stroma score) of TCGA colon cancer samples. High-stroma-content: stroma score > median; low-stroma-content: stroma score < median.

To verify the association of the identified transcriptional signatures with CTS, we analyzed the TCGA colon cancer dataset. We divided these cancers into high-stroma-content and low-stroma-content groups on the basis of their intratumoral stromal content evaluated by ESTIMATE [19] and found that 153 upregulated genes in CTS had significantly higher expression levels in the high-stroma-content group than in the low-stroma-content group. These genes included 18 hub genes in the PPI network of DEGs and 6 TFs, 40 kinases, and 12 MMTRs encoding genes that regulated the DEGs (Supplementary Figure S5, Table S9). We also found 27 downregulated genes in CTS which had significantly lower expression levels in the high-stroma-content group, including 14 hub genes, and genes encoding 2 TFs, 18 kinases, and 3 MMTRs (Supplementary Figure S5, Table S9). Interestingly, most of the downregulated hub genes in CTS were also downregulated in the high-stroma-content colon cancers (Supplementary Figure S5). These results indicate that many transcriptional signatures of CTS identified by the meta-analysis are tumor stroma-specific. In addition, we found that CD8+ T cells had significantly higher enrichment levels in CTS versus colon normal stroma (Student's* t*-test,* p*=0.016), as well as in the high-stroma-content colon cancers versus the low-stroma-content colon cancers (Student's* t*-test,* p*=3.3*∗*10^−8^). It indicates that CD8+ T cells tend to have elevated infiltration in the TME of colon cancer. Interestingly, we found that the higher enrichment levels of CD8+ T cells were associated with better DFS in the low-stroma-content colon cancers, but not in the high-stroma-content colon cancers (Figure 7). It suggests that the immune cells exert an antitumor effect only when they have infiltrated into tumor cells and that the immune cells in the tumor stroma may not have such a direct antitumor effect.

This study has identified a number of CTS-associated transcriptional signatures that could be biomarkers for colon cancer diagnosis and prognosis and may provide therapeutic targets for colon cancer. However, to translate these findings into clinical application, further experimental and clinical validation would be necessary.

## 5. Conclusions

The identification of CTS-specific transcriptional features may provide insights into the mechanism that mediates the development of colon cancer and thus has potential clinical implications for colon cancer diagnosis and treatment.

## Data Availability

The datasets (GSE31279, GSE35602, and GSE46824) were downloaded from the NCBI GEO database (https://www.ncbi.nlm.nih.gov/geo/), the TCGA colon cancer dataset was downloaded from the website https://portal.gdc.cancer.gov/, and the colon metabase dataset was from SurvExpress (http://bioinformatica.mty.itesm.mx/SurvExpress).

## Abbreviations

CTS: Colon tumor stroma
DEGs: Differentially expressed genes
PPI: Protein-protein interaction
ES: Effect size
TME: Tumor microenvironment
ECM: Extracellular matrix
TFs: Transcription factors
MMTRs: Master metabolic transcription factors
OS: Overall survival
DFS: Disease-free survival
CRC: Colorectal cancer
GEO: Gene Expression Omnibus
TCGA: The Cancer Genome Atlas
FDR: False discovery rate
GSEA: Gene-set enrichment analysis
FFM: Function-first module
CFM: Connection-first module.

## Supplementary Materials

Supplementary Materials Supplementary Figure S1: illustration of PCA and density plots as validation tools for batch effect removal. Plot of principal components: (A) before batch effect removal and (B) after batch effect removal. Plot of density: (C) before batch effect removal and (D) after batch effect removal. The multidimensional scaling of the datasets demonstrates that, before the batch effect adjustment, each dataset obviously separated from all the others, whereas, after batch effect adjustment, samples from all the datasets are incorporated clearly. Supplementary Figure S2: six KEEG pathways that are significantly associated with the downregulated genes in colon tumor stroma relative to normal stroma. Supplementary Figure S3: the genes (DEGs and their upstream regulators) whose expression is significantly associated with disease-free survival in colon cancer (log-rank test,* p*<0.05). Supplementary Figure S4: patients divided into the high-risk group and the low-risk group based on the prognostic gene signatures identified. A. Overall survival. B. Disease-free survival. Supplementary Figure S5: numbers of overlapping genes between the DEGs between colon tumor stroma and normal stroma and their upstream regulators and the DEGs between high-stroma-content and low-stroma-content colon cancers. UP TCGA: upregulated differentially expressed genes between high-stroma-content and low-stroma-content TCGA colon cancer samples and DOWN TCGA: downregulated differentially expressed genes between high-stroma-content and low-stroma-content TCGA colon cancer samples. Supplementary Table S1: a summary of the datasets used in this study. Supplementary Table S2: upregulated genes in colon tumor stroma versus colon normal stroma. Supplementary Table S3: downregulated genes in colon tumor stroma versus colon normal stroma. Supplementary Table S4: the top 10 upregulated and top 10 downregulated genes in colon tumor stroma. Supplementary Table S5: 44 KEGG pathways that were significantly associated with the upregulated genes in colon tumor stroma (CTS). Supplementary Table S6: upstream transcription factors and kinases regulating the differentially expressed genes between colon tumor stroma and normal stroma. Supplementary Table S7: master metabolic transcriptional regulators (MMTRs) (iRegulon normalized enrichment score NES > 3.0) regulating the differentially expressed genes between colon tumor stroma and normal stroma. Supplementary Table S8: hub genes in the protein-protein interaction network of the differentially expressed genes between colon tumor stroma and normal stroma. Supplementary Table S9: overlapping genes between the DEGs between colon tumor stroma and normal stroma and their upstream regulators and the DEGs between high-stroma-content and low-stroma-content colon cancers.
