# Supplementary material for: Identification of Transcriptional Signatures of Colon Tumor Stroma by a Meta-Analysis
Source: J Oncol. 2019 May 2;2019:8752862. doi: 10.1155/2019/8752862 (PMC6521457; doi:10.1155/2019/8752862)
Supplement: Supplementary Materials — Supplementary Figure S1: illustration of PCA and density plots as validation tools for batch effect removal. Plot of principal components: (A) before batch effect removal and (B) after batch effect removal. Plot of density: (C) before batch effect removal and (D) after batch effect removal. The multidimensional scaling of the datasets demonstrates that, before the batch effect adjustment, each dataset obviously separated from all the others, whereas, after batch effect adjustment, samples from all the datasets are incorporated clearly. Supplementary Figure S2: six KEEG pathways that are significantly associated with the downregulated genes in colon tumor stroma relative to normal stroma. Supplementary Figure S3: the genes (DEGs and their upstream regulators) whose expression is significantly associated with disease-free survival in colon cancer (log-rank test, p<0.05). Supplementary Figure S4: patients divided into the high-risk group and the low-risk group based on the prognostic gene signatures identified. A. Overall survival. B. Disease-free survival. Supplementary Figure S5: numbers of overlapping genes between the DEGs between colon tumor stroma and normal stroma and their upstream regulators and the DEGs between high-stroma-content and low-stroma-content colon cancers. UP TCGA: upregulated differentially expressed genes between high-stroma-content and low-stroma-content TCGA colon cancer samples and DOWN TCGA: downregulated differentially expressed genes between high-stroma-content and low-stroma-content TCGA colon cancer samples. Supplementary Table S1: a summary of the datasets used in this study. Supplementary Table S2: upregulated genes in colon tumor stroma versus colon normal stroma. Supplementary Table S3: downregulated genes in colon tumor stroma versus colon normal stroma. Supplementary Table S4: the top 10 upregulated and top 10 downregulated genes in colon tumor stroma. Supplementary Table S5: 44 KEGG pathways that were significantly associated with [file 8752862.f1.zip › supplemetary figures.docx]

**Figure S1**

**
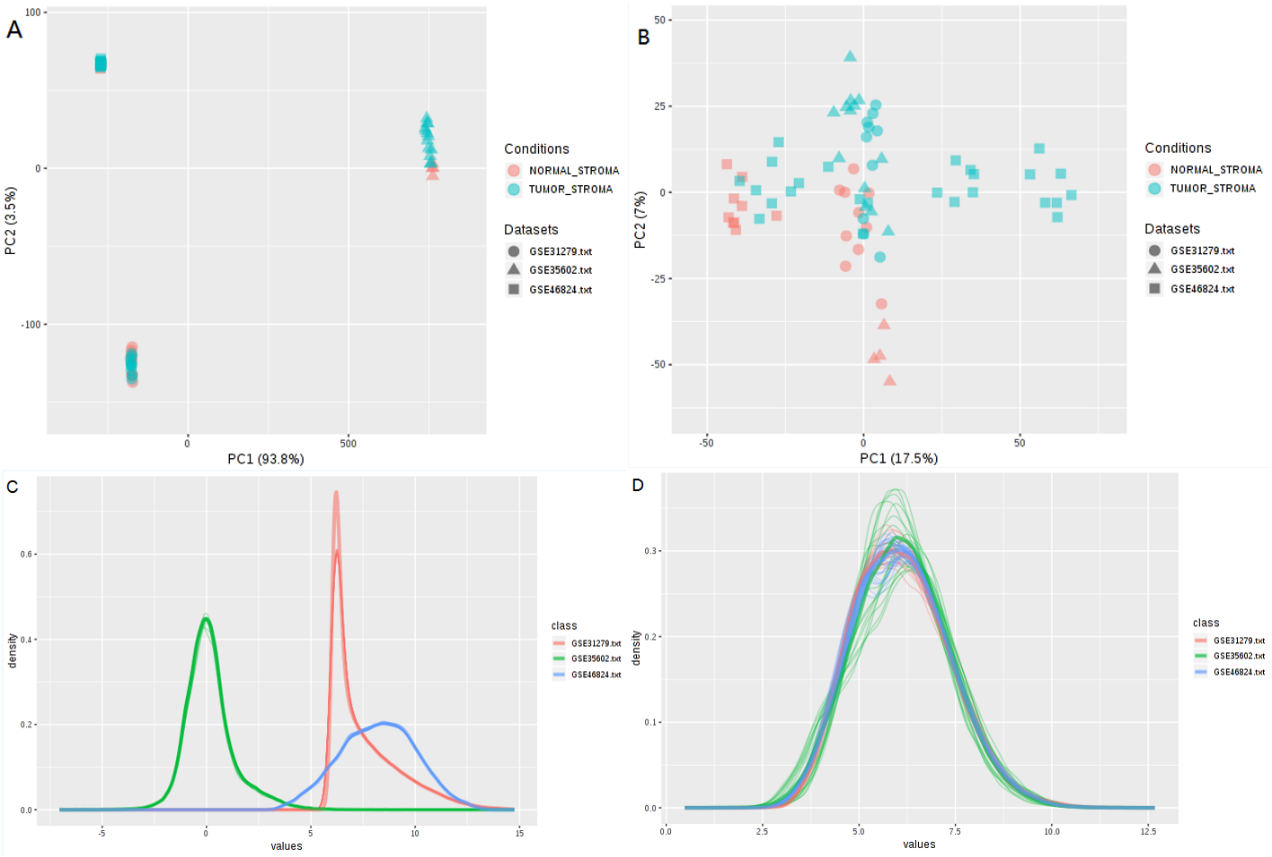
**

**Figure S1.** Illustration of PCA and density plots as validation tools for batch effect removal. Plot of principal components: (A) before batch effect removal, and (B) after batch effect removal. Plot of density: (C) before batch effect removal and (D) after batch effect removal. The multidimensional scaling of the datasets demonstrates that before the batch effect adjustment, each dataset obviously separated from all the others, whereas after batch effect adjustment, samples from all the datasets are incorporated clearly.

**Figure S2.**

**Figure S2.** Six KEEG pathways that are significantly associated with the downregulated genes in colon tumor stroma relative to normal stroma.

**Figure S3.**

**
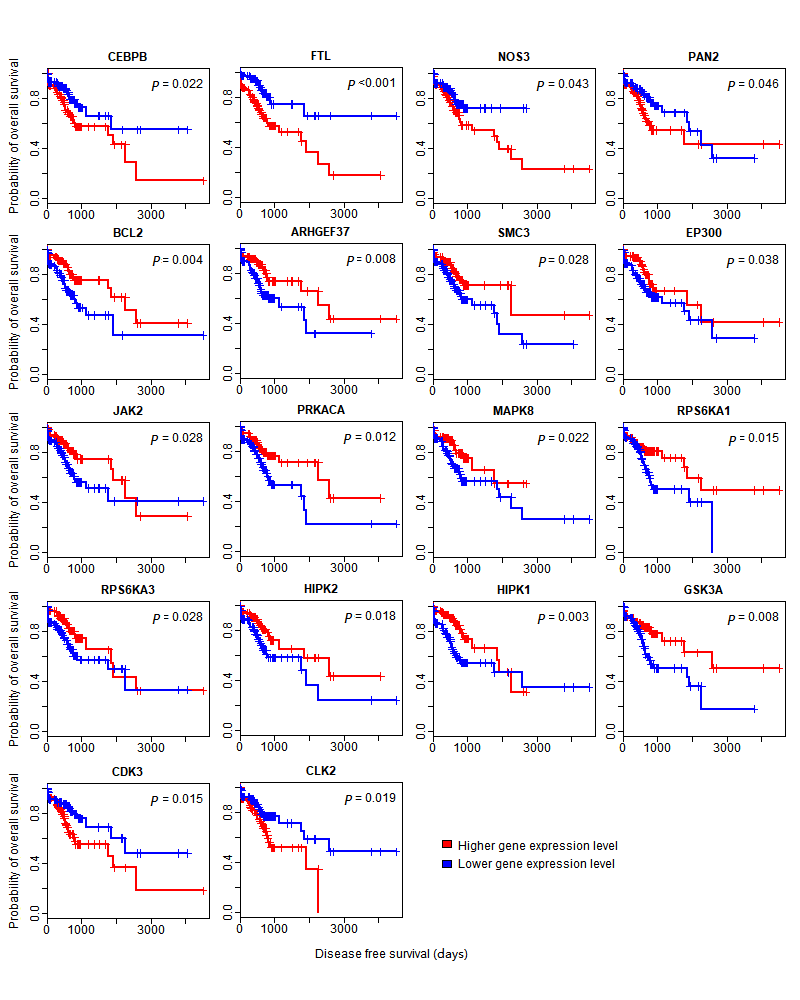
**

**Figure S3.** The genes (DEGs and their upstream regulators) whose expression is significantly associated with disease-free survival in colon cancer (log-rank test, *p*<0.05).

**Figure S4**.


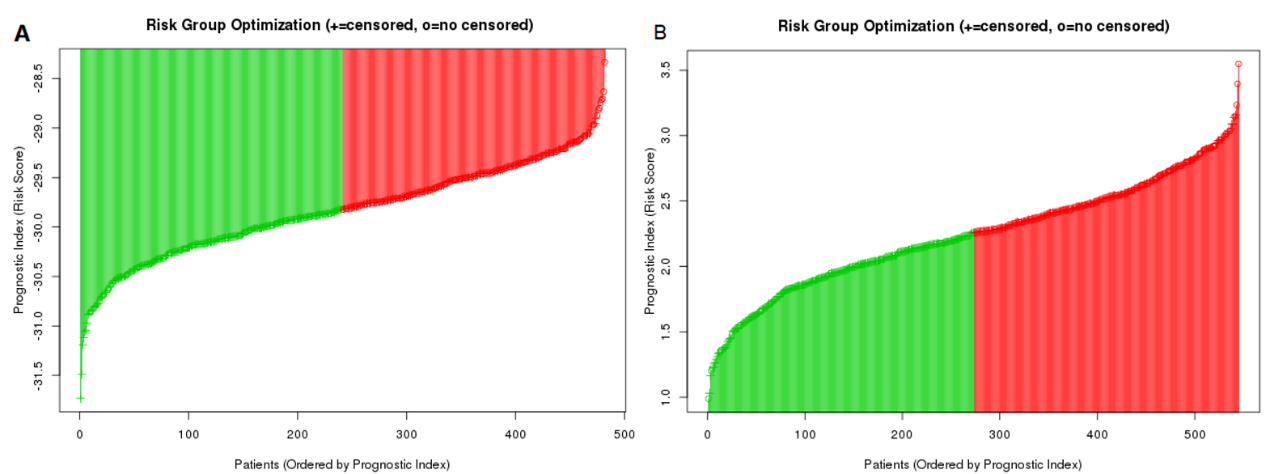


**Figure S4**. Patients divided into the high-risk group and the low-risk group based on the prognostic gene signatures identified. A. Overall survival. B. Disease-free survival.

**Figure S5**.


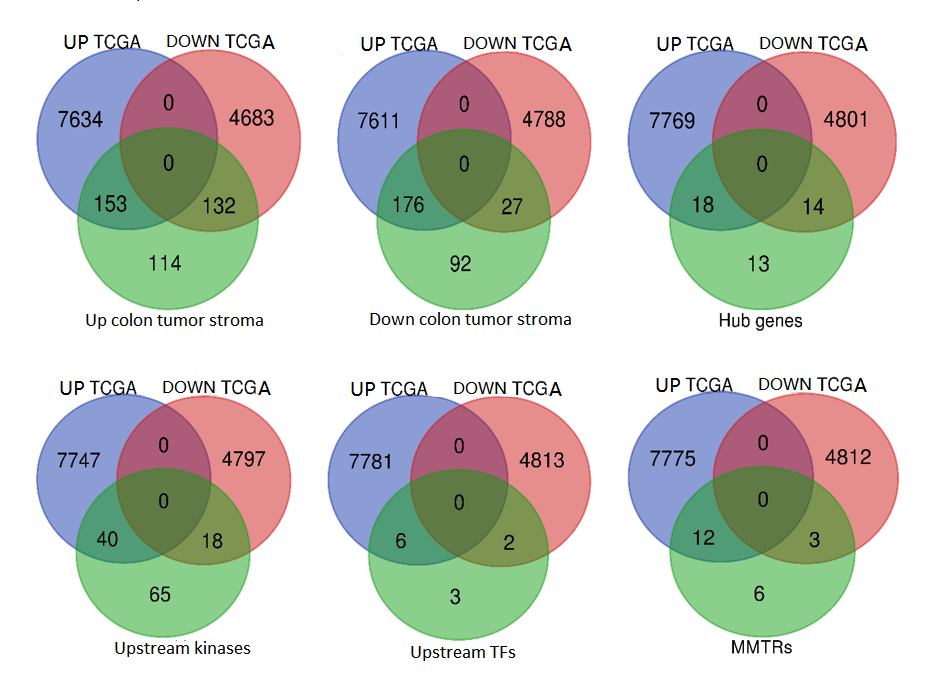


**Figure S5**. Numbers of the overlapping genes between the DEGs between colon tumor stroma and normal stroma, and their upstream regulators, and the DEGs between high-stroma-content and low-stroma-content colon cancers. UP TCGA:Upregulated differentially-expressed genes between high-stroma-content and low-stroma-contentTCGA colon cancer samples and DOWN TCGA:Downregulated differentially-expressed genes between high-stroma-content and low-stroma-content TCGA colon cancer samples.
